# Analysis of the level of polypharmacy in patients from an isolated rural area: effect of age, sex, and chronic diseases

**DOI:** 10.3389/fdgth.2025.1508505

**Published:** 2025-06-10

**Authors:** Susana Abdala Kuri, Chaxiraxi Morales, Alexis M. Oliva, Adama Peña, Sandra Dévora

**Affiliations:** ^1^ Departamento de Medicina Física y Farmacología, Facultad de Farmacia, Universidad de La Laguna, Tenerife, Spain; ^2^ Departamento de Ingeniería Química y Tecnología Farmacéutica, Facultad de Farmacia, Universidad de La Laguna, Tenerife, Spain

**Keywords:** DRP, NOM, polypharmacy, safety, rural area

## Abstract

**Introduction:**

The increase in life expectancy and the greater number of chronic diseases have led to a greater use of medications. This polypharmacy can cause a greater number of drug-related problems and negative results on the patient's health associated with medication, which is why most health services are focused on solving these problems. Machine learning uses different techniques to generate knowledge in health, one of them is regression, whose model establishes that a prognosis is created from a dependent variable and a series of independent variables.

**Materials and methods:**

Data collection was conducted during 2021–2022 in an isolated rural pharmacy. The screening of participants susceptible to being part of the study began at the time of dispensing, verifying that they were part of the personalized dosing system (PDS) service.

**Results:**

The study population consisted of 78 participants, predominantly female. The sociodemographic profile was characterized by being female, between 66 and 80 years of age. The number of chronic diseases per participant was 4.25 ± 1.49. During the study phase, a total of 450 drug-related problems (DRPs) were detected, with an average of 5.64 ± 2.69 DRPs per participant.

**Discussion:**

Age and the assigned polypharmacy level are the factors that most influence the final polypharmacy level. However, it is necessary to include the variable “chronic diseases” since in some situations it seems to be significant.

**Conclusion:**

The factors that most influence the polypharmacy index are patient age and initial polypharmacy level and, to a lesser extent, but no less important, the number of chronic diseases.

## Introduction

There has been an increase in life expectancy in recent years characterized by a greater number of chronic diseases and greater multimorbidity, which increases the consumption of multiple medications (1–3). This polypharmacy, inherent to chronic diseases, is associated with the inappropriate use of medications and other problems such as lack of therapeutic adherence, increased risk of side effects, and drug interactions (4, 5).

The onset of chronic diseases is a gradual process (6) and requires pharmacological treatment for its control, which is proportional to the number of pathologies. In Spain, it is estimated that >77.6% of patients over 65 years of age have at least one chronic disease (7). This polypharmacy, essential for the control of pathologies, can lead to the appearance of DRPs. According to the Pharmaceutical Care Network Europe Association, a drug-related problem (DRP) is defined as “an event or circumstance involving a pharmacological therapy that actually or potentially interferes with desired health outcomes” (8).

The causes of failure in the control of chronic pathologies seem to lie in fragmented care and a healthcare organization focused on acute and hospital patients rather than on healthcare that guarantees continuity of care (9) in chronic patients. This failure increases polypharmacy and with it, the prevalence of DRPs and/or negative outcomes associated with medication (NOM). The Guide to Professional Pharmaceutical Assistance Services defines NOM as “negative results in the health of the patient, not appropriate to the objective of pharmacotherapy, associated or that may be associated with the process of use of the drugs” (10).

Medication review is a professional pharmaceutical service that contributes to reducing the number of DRPs and NOMs present in the patient (11). Community pharmacists are the most accessible healthcare professionals in the system. They are responsible for the distribution of medicines and are also the last healthcare professionals who are in contact with the patient before taking the medication. On the other hand, community pharmacists contribute to the provision of professional pharmaceutical care services, which reduces the prevalence of DRP or NOM.

These professional pharmaceutical services are defined as “care activities provided from the community pharmacy by a pharmacist who uses his or her professional skills to prevent diseases and improve the health of both the population and the recipients of medicines” (10).

Spain has more than 22,000 community pharmacies, where pharmacists play a crucial role in health advice (12) and “ensure compliance with the guidelines established by the doctor responsible for the patient and cooperate with him or her in the monitoring of his or her treatment through pharmaceutical care procedures” (13).

The new healthcare models need to be focused on an innovative and multidisciplinary approach that responds to individual needs. Pharmacists are responsible for dispensing medications (13) and as part of the national health system, they share the mission of guaranteeing the safe and effective use of medications (10).

The prevalence of chronic diseases and polypharmacy requires the development of studies to detect DRPs and/or NOMs and their resolution in order to improve the control of health problems and optimize healthcare resources (10, 14). This research must be characterized by being predictive, participatory, personalized, and preventive (15).

Machine learning uses different techniques to generate health knowledge that allows grouping, classifying, or creating decision trees before the patient presents the disease or predicts its evolution. One of the techniques it uses is regression, whose model focuses on creating a prognosis from a dependent variable and a series of independent variables (16, 17).

Different authors have used linear regression in studies of scientific health interest, such as Nagino et al. (18) who validated a dry eye symptom stratification algorithm, where patients can be classified based on the symptom in seven different categories. Iwendi et al. (19) designed an algorithm consisting of two parts, namely, AdaBoost and Random Forest, which predicts the impact of COVID-19 on patients based on multiple factors, for example, sociodemographic profile or international travel. On the other hand, Yuan et al. (20) created a tool based on the medical data recorded by patients with lung cancer that allows their survival to be estimated; a methodology similar to that used by Zong et al. (21) who, through registered medical information, created a classification of the different primary cancers that can even predict primary oncological stages without diagnosis.

In Spain, Reyes et al. (22) developed a tool that identifies the patient's profile in relation to their disease, failed back syndrome, and their attitude to taking medication. This tool guides the most effective health strategy to promote the rational use of medication, as did Vendrusculo et al. (23) who validated an equation model that relates psychosocial aspects, biomarkers, and pain perception in patients with rheumatoid arthritis.

Statistical analysis facilitates the development of tools from patient health data, establishing causal relationships and evaluating the effectiveness of interventions by health professionals, leading to more personalized medicine and optimization of health resources.

Different models have been developed from a statistical analysis. The statistical analysis of the linear model must comply with the following two premises: the errors are normally distributed with zero mean and constant variance, and the dependent variable is linearly related to the independent variables; however, when this is not possible, it can be solved by transforming the response variable (e.g., taking logarithms), although these transformations do not always manage to correct one or more of the premises that condition the linear models. The use of generalized linear models (GLMs) is an alternative to the transformation of the dependent variable/response and the lack of normality (24).

Generalized linear models (GLMs) (21) are an extension of linear models that allow the use of non-normal error distributions (binomial, Poisson, gamma, etc.) and non-constant variances. A GLM model is a model that links responses (“dependent” variables) to other “independent” or “explanatory” variables.

Capasso et al. (25) conducted a study during the COVID-19 pandemic, where they analyzed the patient's sociodemographic profile, alcohol consumption, and mental health, by means of a multivariate analysis using GLMs. The main conclusion of the study was that the probability of consuming alcohol was higher in men and in people with symptoms of depression and anxiety.

The main objective of the present study was to analyze the level of polymedication of an isolated rural population in a municipality on the island of Santa Cruz de Tenerife, Spain. In this case, the possible effects of the age, sex, and number of chronic diseases suffered by the participant were analyzed. To do this, a GLM model was used to identify and determine the effect of the different factors mentioned above.

## Materials and methods

### Statistical model

The first step in applying GLM models is to explore the data, specifically, to understand the nature of the data in order to determine the most appropriate link or canonical function. Once the data have been extrapolated, the most appropriate model is selected.

Poisson regression models are the most appropriate ones for modeling events in which the results are counted. The *Yi* response is a value that follows the Poisson distribution. The logarithm of the expected values (mean) is assumed to be linearly modeled using the logarithm of the Poisson regression as the link function (21). For this reason, a Poisson regression model is also called a log-linear model. Based on the above, a Poisson regression model is proposed, characterized by:

(1) log(E(Yi))=log(μi)=β0+β1xi1+β2xi2+…+βpxip

where each *β* represents the effect of the corresponding predictor variable.

Regarding the interpretation of these coefficients, the relative risk (RR) is represented by the incidence rate of the events associated with an increase of one unit in the covariate *x_i_*.

In this case, the RR is used, which increases when RR > 1 and decreases when RR < 1. The risk is the same when RR = 1.

In the present case, it is a multifactorial analysis since it is composed of at least two explanatory variables (one continuous and one categorical).

The relationship of variables analyzed here is summarized as:

- Med = number of medications taken by patient Xi (continuous-discrete variable)
- Age = age (years) of the patient (continuous-discrete variable)
- Sex = sex of the patient (male or female, categorical variable)
- Chr dis = number of chronic diseases declared by the patient (continuous-discrete variable)
- Help = type of help received by the patient (categorical variable; two levels: 0 = does not receive help; 1 = receives some type of help)
- Pol level = level of polypharmacy assigned to the patient (categorical variable; four levels): level 1 (<7 medications), level 2 (8–10 medications), level 3 (11–13 medications), and level 4 (>14 medications)
- Cog Cap_ = cognitive capacity of the patient (categorical variable)
- DRP = drug-related problems (discrete-continuous variable)
- NOM = negative outcomes associated with medication (discrete-continuous variable)

DRPs and NOMs have been classified according to the Guide to Professional Pharmaceutical Assistance Services (10).

Data analysis was performed using the freely available R program (http://www.r-program.org). Appendix 1 shows the subroutine used in this study for the R program.

The suitability of the model was assessed using the deviance, which allows the variability of the model to be quantified. To do this, the deviance of the null model (null deviance) was compared with the residual deviance (residual deviance). The expression to be used is the following:

(2) $$D^{2}=\frac{\mathrm{Null}\;\mathrm{model}\;\mathrm{deviance}-\mathrm{Residual}\;\mathrm{deviance}}{\mathrm{Null}\;\;\mathrm{model}\;\;\mathrm{deviance}}*100$$

The Shapiro–Wilk test allowed the normality of the data to be verified.

### Study population

This study was a descriptive, prospective, and longitudinal study of a sample of chronic patients, who were regular users of the rural pharmacy who were part of the personalized dosing systems service. Patients included in the personalized dosage system (PDS) service must be polymedicated patients over 18 years of age. The inclusion criteria were as follows: patients over 18 years of age with five or more chronic drugs.

Data were collected during 2021–2022 and screening of patients eligible to participate in the study was carried out at the time of dispensing their usual medication.

The inclusion criteria were:

- Patients over 18 years of age and belonging to one of the target populations to be evaluated by the research pharmacist:

- Patients who presented some DRPs
- Patients with constant changes in their treatment plan
- Patients with risk factors for NOMs
- Patients with more than five chronically used medications

The exclusion criteria were:

- Patients undergoing chemotherapy treatment
- Patients who, despite being users of the PDS, continue to have poor therapeutic adherence
- Patients who do not sign the informed consent for data transfer

### Material resources

- Personalized care area: space within the pharmacy separated from the dispensing area, which guarantees an atmosphere of trust and confidentiality with the patient.
- Preparation and repackaging area: space suitable for preparing PDS, in which no other activity can be carried out simultaneously with blister packaging.
- Storage area: space dedicated to storing each patient's medications.
- Basic material necessary for the preparation of PDS: blister, dosing systems service in handling material, protective material, roller, and labels for identifying the dosing systems service.
- Medication review sheet: registration model where the patient's treatment plan and the most relevant data on taking the medication are recorded, as well as the observations that allow the patient's initial status to be completed. The Dader method was used to conduct the personal interview. This method consists of different stages where the safety, effectiveness, and need for pharmacological treatment are evaluated. This method detects DRP or NOM. The methodology is included in the Guide to Professional Pharmaceutical Assistance Services (10).
- BotPlus: drug database of the General Council of Official Colleges of Pharmacists and source of obtaining complete and updated information on drugs and health products marketed in Spain.
- NausiSPD: computer software used to create dosing systems service.

### Ethical aspects

The study was conducted after the approval of the Research Ethics Committee of the Hospital Universitario de Canarias with the code SPD-SFT2122 and in accordance with the ethical principles for medical research on human beings expressed in the Declaration of Helsinki.

The treatment and transfer of personal data of the participants was adapted to the provisions of the Spanish Organic Law 3/2018, of December 5, on the Protection of Personal Data and Guarantee of Digital Rights.

The study guarantees the complete dissociation of the participants' personal data with the information obtained at the end of the research, so that the said final information, property of the research pharmaceutical company, cannot be linked in any way to the identification of a specific participant with the aim of knowing their identity.

No participant should be included in the study without first giving their written informed consent for the transfer of data. Each participant has sufficient time to read, understand, and sign the explanations of the said informed consent, in addition to receiving a copy of the signed document.

## Results

**Figure 1:**
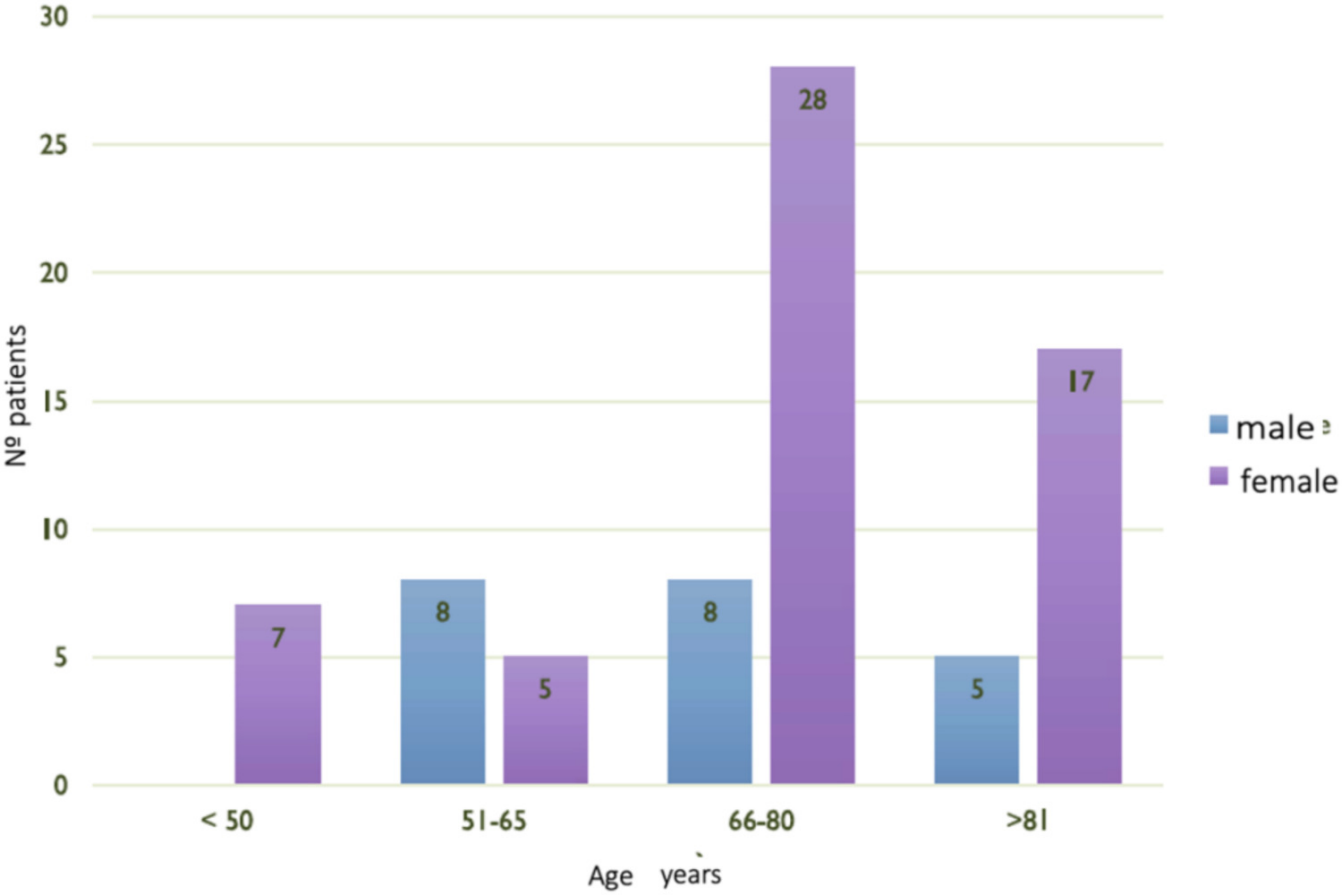
Sociodemographic profile.

The study population consisted of 78 participants, with a predominance of females (73.08%) over males (26.92%), with an average age of 72.49 ± 13.92 years. Participants were stratified according to sex and age (Figure 1).

The sociodemographic profile was characterized by being a woman aged between 66 and 80 years old, living with her spouse or family member, and having normal cognitive ability. Regarding the help received to manage medication, 50% of the study population needed help to manage medication from a family member or caregiver.

The number of chronic diseases per participant was 4.25 ± 1.49, with polymedication levels 2 and 3 being the most frequent (Table 1). From the study sample, the maximum number of medications per patient was set at 20 medications. This patient was a woman aged between 51 and 65 years who suffered from refractory arterial hypertension with poor control, depression, hypercholesterolemia, and an amputation of a lower limb.

**Table 1:** Stratification by sex, age, and level of polypharmacy.

Levels of polypharmacy	Male				Female			
	1	2	3	4	1	2	3	4
<50 years					3	1	2	1
51–65 years	5	1		2		1	2	2
66–80 years	3	4	1		1	7	11	9
>81 years	1	2	1	1	1	5	11	

During the study phase, a total of 450 DRPs were detected, with an average of 5.64 ± 2.69 MRPs per participant, and there were 161 NOMs in 71.79% of the study population associated with these MRPs. The characterization of these DRPs and NOM was published in 2024 by Morales et al. (26) where the prevalence of DRPs and differences for the group of patients were described, after receiving the Cinfa award for the best work of DRP.

**Figure 2:**
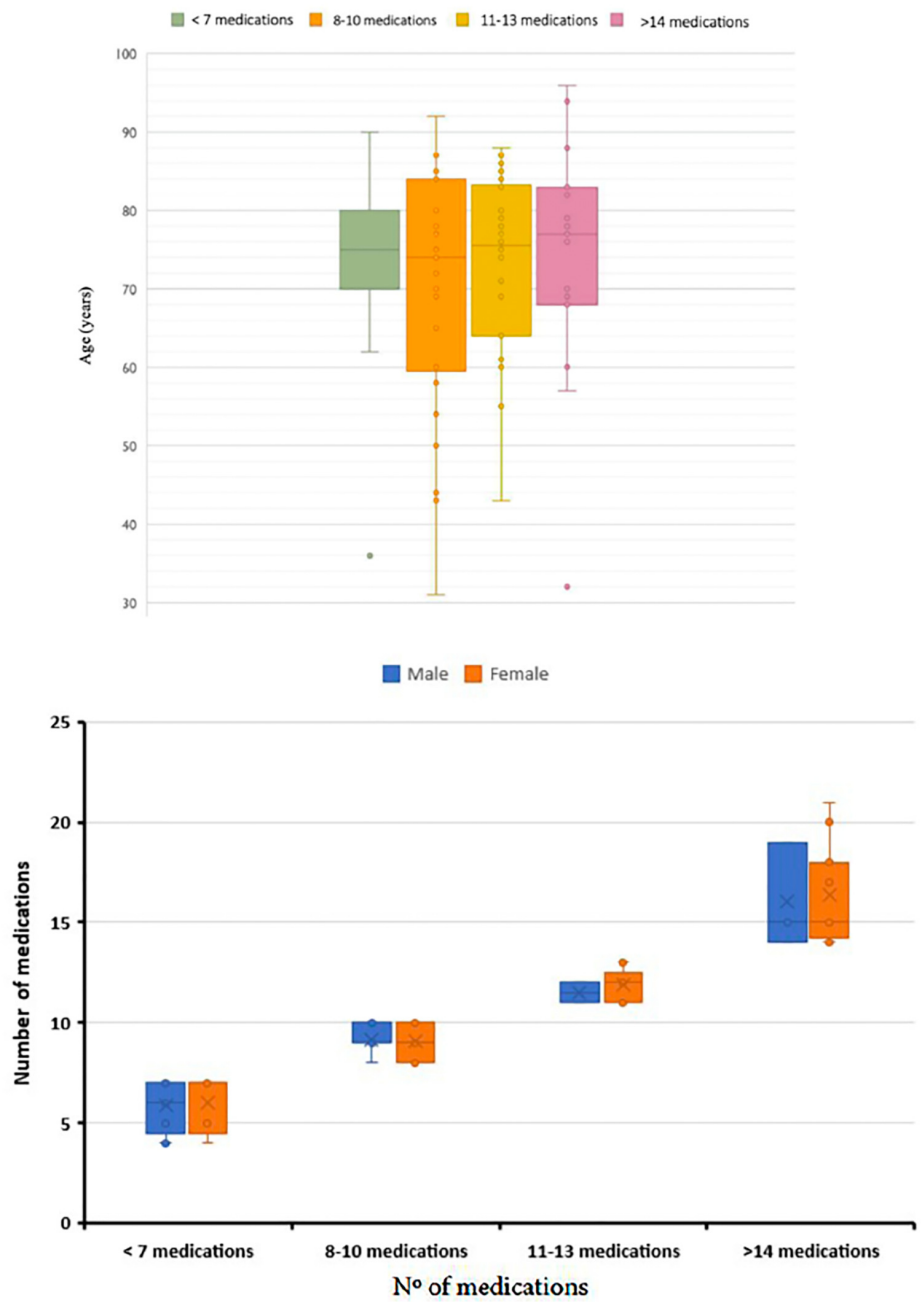
Box plot representation of the different groups of participants classified according to their level of polypharmacy and age (upper) and sex (lower).

The statistical analysis of the data established that the sample of the study population was made up of 75 of the 78 participants who were part of it, since three of them were under 40 years of age, so the values ​they presented were atypical as they exceeded the expected variance (Figure 2).

**Figure 3:**
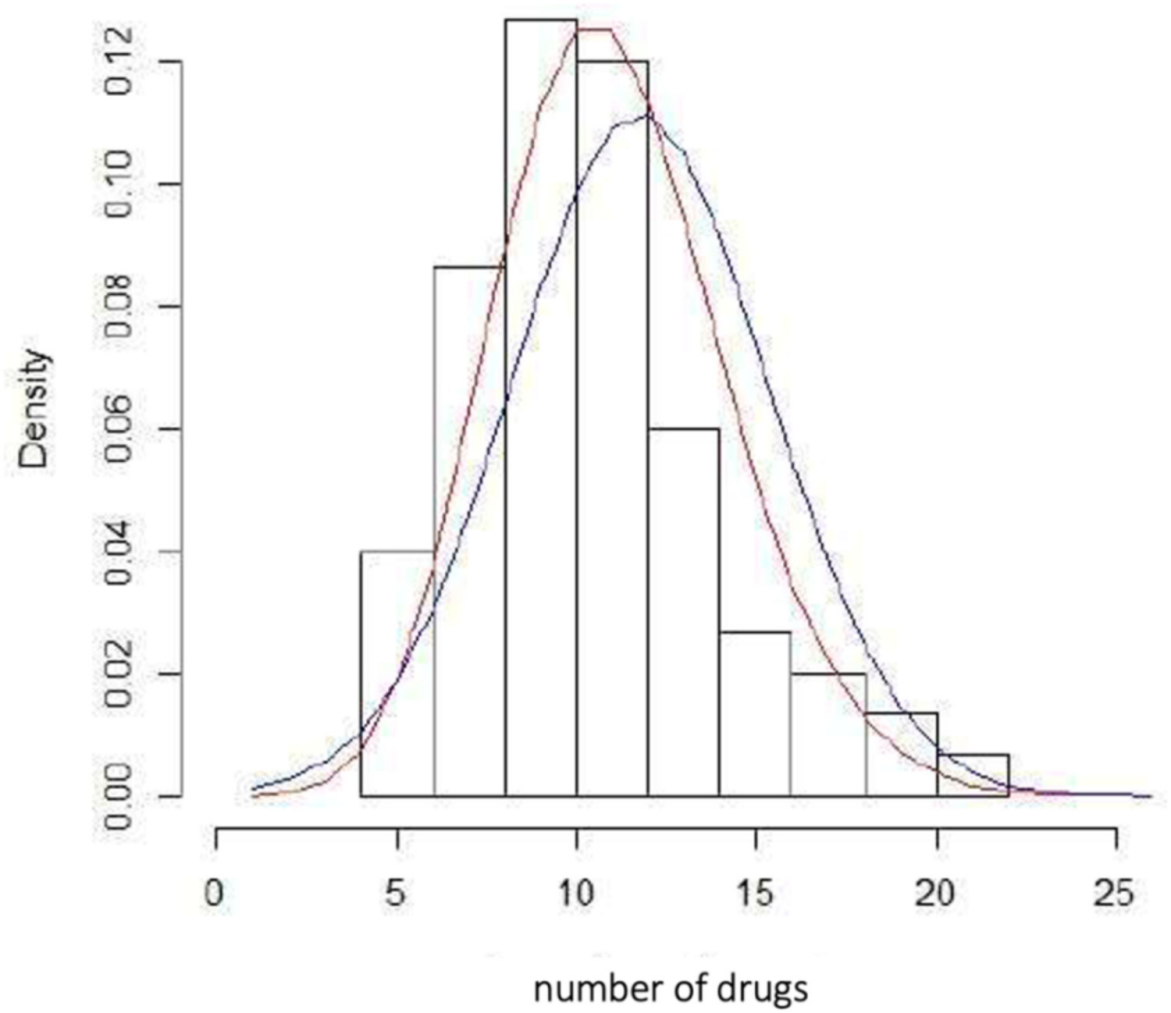
Histogram of relative frequencies of the number of drugs and different distribution functions. Blue (normal distribution), red (Poisson distribution).

Exploration of the data showed that the distribution function that best fitted the data was a Poisson distribution (Figure 3).

Table 2 shows the results of the analysis of variance (ANOVA) and the parameters of the estimated model, using the GLM model with the Poisson link function (Appendix 1).

**Table 2:** Estimated parameters for the analyzed model using the glm() function with a “Poisson” link function.

Coefficients	Estimated	2.5%	97.5%	pro(>*F*)
Intercept	1.260	0.7021	1.8137	<0.001*
Age	2.385 × 10^−3^	−0.0037	0.0086	0.450
Sex	3.3743 × 10^−5^	−0.1659	0.1696	1.000
Chr dis	1.606 × 10^−2^	−0.0276	0.0593	0.469
Help	−1.169 × 10^−2^	−0.1692	0.1453	0.884
Pol level	0.292	0.1955	0.3894	<0.001*
Cog cap_	1.893 × 10^−2^	−0.0639	0.1005	0.652
NOM	−3.611 × 10^−3^	−0.0452	0.0378	0.865
DRP	6.558 × 10^−3^	−0.0236	0.0366	0.669

* Significance for *p* < 0.05.

**Figure 4:**
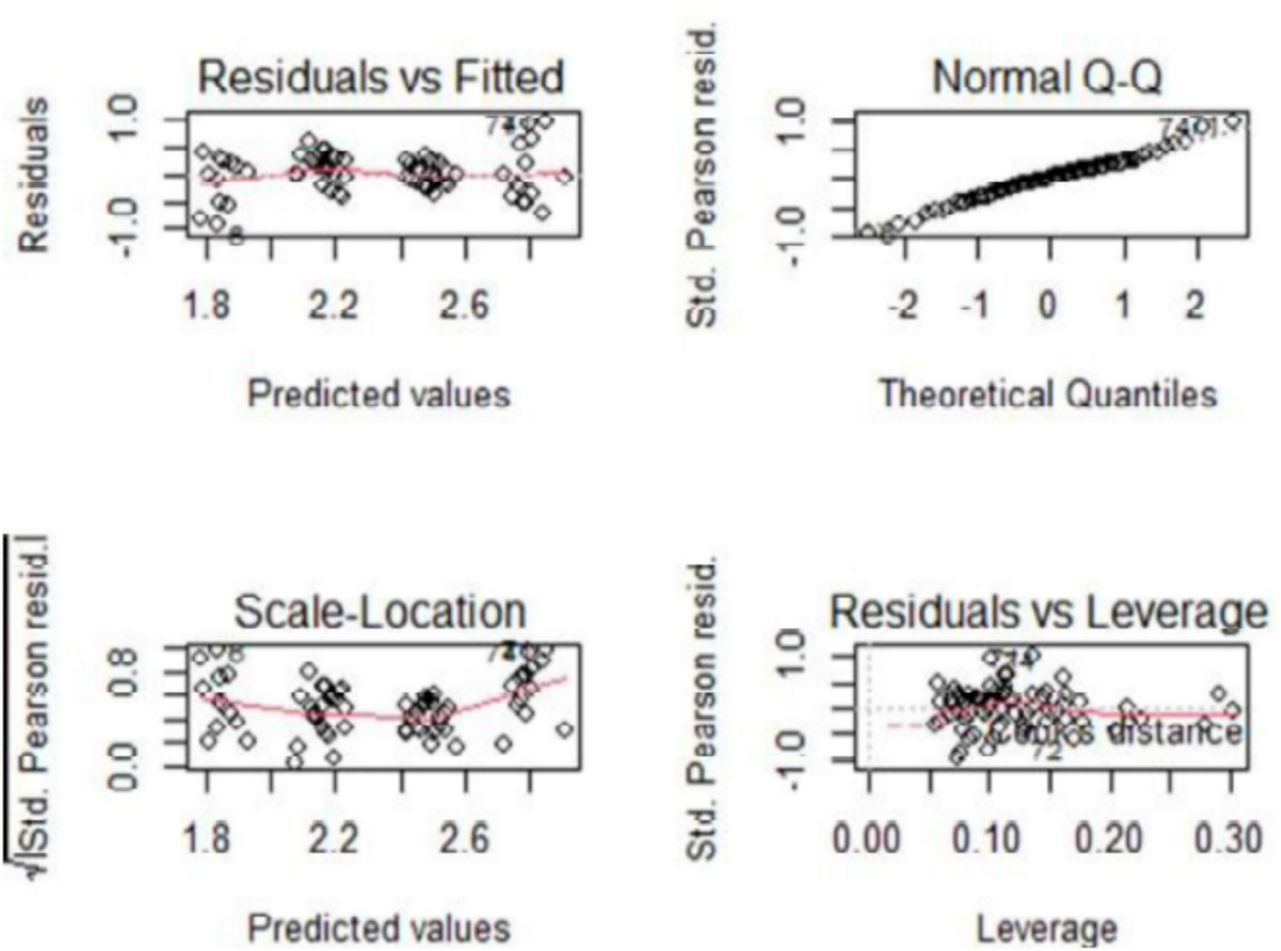
Normality and evaluation of data.

The graphical representations generated by the R program confirmed that the behavior of the residuals and the assessment of data normality fitted a generalized linear model assuming a Poisson distribution (Figure 4).

The goodness of fit of the model was 89.56%, that is, 89.56% of the results were explained by the proposed model. In addition, the Shapiro–Wilk test confirmed the normality of the data (*p* = 0.4168).

In the case of Poisson data, the mean must be equal to the variance; if the data do not meet this hypothesis, the standard errors of the parameter estimators will be incorrect. In these cases, a dispersion parameter, *φ*, can be introduced so that Var(*Y*) = *φµ* (*φ* = 1 indicates being in the Poisson case), the parameter can be calculated as the deviance of the model divided by the degrees of freedom of the residuals of the model.

If the deviance is greater than the degrees of freedom, then there is excessive dispersion or so-called overdispersion. This means that the true variance is much greater than that predicted by the model, and hypothesis tests will find differences that do not exist. There are several reasons for the presence of overdispersion, such as the assumed distribution not being correct; there is heterogeneity due to some known cause, an incorrectly specified model (important variables omitted in the model), outliers, etc. On the contrary, if this ratio is less than one, this is referred to as underdispersion, which is associated with overparameterization.

In the present study, the deviance obtained was 86.48%, and the degrees of freedom of the model were 74, so the parameter phi was >1 (*φ* = 1.17), indicating that there was overdispersion of the data. In this situation, alternatives must be evaluated, for example, using a quasi-Poisson function. In this situation, the model estimates were equal, but the variance was greater in the quasi-Poisson model. Therefore, it was closer to the reality of the dispersion of the data and a correct interpretation of the results.

Table 3 shows the result of applying the glm() function of the R program considering a quasi-Poisson function (Appendix 2).

**Table 3:** Estimated parameters for the model analyzed, using the glm() function with a “quasi-Poisson” link function from the R program.

Coefficients	Estimated	2.5%	97.5%	pro(>*F*)
Intercept	1.260	1.056	1.463	<0.001*
Age	2.385 × 10^−3^	1.224 × 10^−4^	0.004	0.043*
Sex	3.3743 × 10^−5^	−6.120 × 10^−2^	0.061	0.999
Chr dis	1.606 × 10^−2^	9.029 × 10^−5^	0.031	**0** **.** **052**
Help	−1.169 × 10^−2^	−6.938 × 10^−2^	0.045	0.692
Pol level	0.292	0.2566	0.327	<0.001*
Cog cap_	1.893 × 10^−2^	1.131 × 10^−2^	0.049	0.223
NOM	−3.611 × 10^−3^	−1.886 × 10^−2^	0.011	0.643
DRP	6.558 × 10^−3^	−4.489 × 10^−3^	0.017	0.248

Bold values are significant.

* Significance for *p* < 0.05.

## Discussion

The sociodemographic profile was notable for its female predominance, a common characteristic in numerous studies showing that the female sex is more receptive to taking part in observational studies of medications (27–30). The average age was 72.49 ± 13.92 years, being similar for both sexes; of these, 60 participants were over 65 years old and, therefore, considered as being geriatric.

The number of chronic diseases was similar for both sexes, 4.25 ± 1.49 diseases; this prevalence is in the range of results reported by other studies also carried out in Spain, for example, the study by Santos et al. (24) where 61% of the study population had at least six chronic diseases, unlike the study by Pérez et al. where the average number of chronic diseases was 2.5 ± 0.74 (29).

According to ANOVA (data not shown), the level of polypharmacy and number of chronic diseases did not differ: average number of chronic illnesses was 4.25 ± 1.9 (*n* = 16) at polypharmacy level 1, which increased to 4.33 ± 1.53 (*n* = 22) at polypharmacy level 3 and to 4.34 ± 1.51 (*n* = 15) at polypharmacy level 4.

The scientific literature confirms the impact of pharmaceutical interventions not only on reducing polypharmacy but also on the detection of DRPs or NOMs. The distribution of community pharmacies throughout the national territory provides the patient with universal accessibility; therefore, the community pharmacist is the ideal health professional for the detection of DRPs or NOMs. This ability has been amply demonstrated in numerous articles, where pharmacists have detected adverse reactions, drug interactions, prescription errors, etc.

The prevalence of DRPs underscores the need to integrate the community pharmacist into the care team that addresses the patient. In response to the prevalence of DRPs, pharmacists have developed professional pharmaceutical care services whose aim is to promote the rational use of medicines, minimize risk, relieve the healthcare system, and reduce DRPs or NOMs (10).

The most frequent polypharmacy levels were 2 and 3, with 26 and 22 participants, respectively. These results coincide with the previously cited studies. García et al. (27) reported that 44% of their study population took between five and eight medications and 10% took nine or more. On the other hand, the study by Menéndez et al. (28) was characterized by a study population where 70% of the cases took five or more medications for chronic use. Santos et al. (30) found that the prevalence of polymedication in the taking of six chronically used medications was in 93% of the participants in the study.

In relation to the average number of DRPs present in the participants, this was 5.64 ± 2.69 DRPs. When analyzing the prevalence of DRPs based on sex, it was observed that, in the case of being a woman, the prevalence of presenting one DRP was lower than in men, 5.42 ± 2.7 and 6.44 ± 2.60, respectively.

The analysis of the DRPs presented by each participant was performed based on the latest update of the Consensus Document of the Community Pharmacy Pharmaceutical Care Forum (8). In the present study, the percentage of DRPs present in the participants was higher than that found in other publications. The study by Espinoza et al. (31) reported 154 DRPs in 86 participants, as did Villagra et al. (32) who found 151 DRPs present in 31% of the study population. These results show a lower percentage than that observed in the present study, because the latest update of the Consensus of the Community Pharmacy Pharmaceutical Care Forum includes a greater number of DRPs.

The results in Table 2 show that the number of medications depends only on the degree of polypharmacy, as the other variables were not significant (*p* > 0.05). In addition, the dispersion parameter was greater than one (*ϕ* = 1.17), suggesting an overdispersion of the data. In such a scenario, it's necessary to transform the model to the quasi-Poisson function (Table 3). The model that best describes the data presented (Table 4) is defined by the variables “AGE”, “Pol level,” and the effect on the mean (intercept), all of which are significant since *p* < 0.05. However, the authors have considered including the effect of “Chr dis” in their model, although the *p*-value is >0.05 (*p* = 0.052), slightly higher than the significance value considered (*α* = 0.05), whose estimate is different from zero as its 95% confidence intervals do not include zero (see Table 3). The validity of this hypothesis needs to be confirmed in future studies. Therefore, the model defined by the glm() function and the “quasi-Poisson” family is as follows:

(3) $$\begin{array}{r} \mathrm{Med}=\exp(1.260+0.00238\;*\mathrm{AGE}+0.0161*\mathrm{Chr}\;\mathrm{dis}\\ \;\;+\;0.292*\mathrm{Pol}\;\mathrm{level}) \end{array}$$

**Table 4:** Number of medications estimated according to model parameters using the quasi-Poisson link function.

Pol level	65 years and 6 Cro dis	74 years and 6 Cro dis	85 years and 6 Cro dis
#1	6.01	6.19	6.57
#2	8.12	8.30	8.80
#3	10.9	11.1	11.8
#4	14.6	14.9	15.8

The analysis of the model allows us to deduce that the RR is the same for the variables “AGE” (RR = 1.002) and “Chr dis” (RR = 1.016) since RR is practically equal to 1, while for the variable “Pol level,” the risk rate is >1 (RR = 1.339). Therefore, the risk of increasing the number of medications increases by one unit for each year and new chronic disease that the participant has, while the RR by medication level increases by 33% more than the previous two (Table 4).

Regardless of age or chronic diseases, each participant takes an average of 3.52 medications [exp(intercept)], that is, the value estimated based on the ordinate of the model when exp(Bo).

Suppose a patient aged 74 with six declared chronic diseases (these values ​correspond to the average participant) takes 6.89 medications, rounded up to seven medications. If this average patient increases their age by 1 year and a new chronic disease is not declared, they will take 9 (predicted value, 9.22) medications, although this effect will be aggravated if during that time a new disease is declared, they will take 11 (predicted value: 11.46) medications. This difference is due to the greater effect of the level of polypharmacy moving from level 2 to level 3. This effect should be more intense if the patient belongs to medication level 3. To analyze this effect, the authors considered the same model and function with the only difference of introducing the level of polypharmacy (Pol level) as a variable with four levels.

In this case, the final model depends only on “AGE” and the effect of each of the levels of polypharmacy. In this model, the value of the ordinate (i.e., intercept) corresponds to level 1 of polypharmacy. To obtain the effect of the rest of the levels, the value of the ordinate must be added to the estimated value for that effect. The deviance obtained in this case was 90.13%, three points above that obtained in the previous model, which gives an idea of the suitability of the model to interpret the results.

As expected, the RR increases with the level of polypharmacy, going from 1.46 for level 2 to 2.53 for level 4. In this model, the variable “Chr dis” is not significant (*p* > 0.05), and the 95% confidence intervals include the value zero.

According to these data, the final model is as follows:

(4) $$\begin{array}{rl} \mathrm{Med}\; & =\exp(1,49+0,0026\cdot \mathrm{AGE}+1,87\cdot \;\mathrm{Pol}\;\mathrm{level}2\\ & +2,12\cdot \;\mathrm{Pol}\;\mathrm{level}3+2,42\cdot .\mathrm{Pol}\;\mathrm{level}4) \end{array}$$

In this case, the number of medications that a patient initially takes and that determines their assigned polypharmacy level does not change significantly with age, since its effect only involves an increase of 1.62% in the number of medications per year and is independent of the number of chronic diseases they suffer from.

For example, a 65-year-old patient with “POL_level” 1 takes 5.26 medications, while if their age were 85 years, the number of medications would be very similar (5.54). This effect is greater in a patient of the same age and Pol level, who goes from taking 1 more medication 20 years later, from 13.2 to 14 medications.

Therefore, it can be summarized that age is the only factor to take into account when analyzing the data together with the assigned polypharmacy level. However, it is necessary to increase the population under study in order to determine whether the variable “chronic diseases” should be taken into account, since in some situations it appears to be significant, although in all cases the probability level is very close to 5% (33).

Furthermore, the number of medications does not increase significantly with the patient's age, and with it the possibility of a jump in the level of polypharmacy. This level of polypharmacy is assigned to the patient based on the medication prescribed according to the pathologies that they suffer from, but it is not altered by the patient's age or the increase in diseases over time.

## Conclusion

Statistical analysis of the data shows that the factors that most influence the polypharmacy rate are patient age and initial polypharmacy level and, to a lesser extent, but no less important, the number of chronic diseases. The function of the final model is capable of explaining 85% of the cases, although it is true that this needs to be verified for future patients. Extrapolation to different population groups or areas is possible if an initial screening is previously carried out to verify the presence of a DRP or NOM and a pharmaceutical intervention is subsequently carried out.

## Data Availability

The original contributions presented in the study are included in the article/Supplementary Material; further inquiries can be directed to the corresponding author.
