# Supplementary material for: Analysis of the level of polypharmacy in patients from an isolated rural area: effect of age, sex, and chronic diseases
Source: Front Digit Health. 2025 Jun 10;7:1508505. doi: 10.3389/fdgth.2025.1508505 (PMC12185413; doi:10.3389/fdgth.2025.1508505)
Supplement: Supplementary file 1 [file Datasheet1.pdf]

Supplementary Material

Abbreviation

Chr.dis: Chronic Diseases

Cop_Cap: Cognitive Capacity

NOM: Negative Outcomes Associated

DRP: Drugs-Related Problems

```
# model_2 corresponds to the analysis of the data with the function glm().

model_2 <- glm(model, family =poisson (link=log), data = data)
anova(model_2)

## Analysis of Deviance Table
##
## Model: poisson, link: log
##
## Response: Med
##
## Terms added sequentially (first to last)
##
##
##           Df Deviance Resid. Df Resid. Dev
## NULL                          74     86.476
## AGE        1    1.559         73     84.917
## Sex        1    3.471         72     81.446
## Chr.dis    1   33.992         71     47.454
## Help       1    0.005         70     47.449
## Pol.level 1   38.006         69      9.442
## Cog_Cap    1    0.229         68      9.214
## NOM        1    0.000         67      9.214
## DRP        1    0.182         66      9.031

summary(modelo_2)

##
## Call:
## glm(formula = Med ~ AGE + sex + Chr.dis + help + POL Level +
##     Cog_Cap + NOM + DRP, family = poisson(link = log), data = data)
##
## Deviance Residuals:
##      Min        1Q    Median        3Q       Max
## -0.97385  -0.21893   0.00023   0.19935   0.88322
##
## Coefficients:
##               Estimate Std. Error z value Pr(>|z|)
## (Intercept)  1.260e+00  2.835e-01   4.446 8.76e-06 ***
## AGE          2.385e-03  3.156e-03   0.756    0.450
## Sex          3.374e-05  8.555e-02   0.000    1.000
## Chr.dis      1.606e-02  2.218e-02   0.724    0.469
```

```
## Help          -1.169e-02  8.023e-02  -0.146    0.884
## Pol.level      2.922e-01  4.945e-02   5.908 3.46e-09 ***
## Cog_CAp        1.893e-02  4.196e-02   0.451    0.652
## NOM           -3.611e-03  2.120e-02  -0.170    0.865
## DRP            6.558e-03  1.536e-02   0.427    0.669
## ---
## Signif. codes:  0 '***' 0.001 '**' 0.01 '*' 0.05 '.' 0.1 ' ' 1
##
## (Dispersion parameter for poisson family taken to be 1)
##
##     Null deviance: 86.4761  on 74  degrees of freedom
## Residual deviance:  9.0314  on 66  degrees of freedom
## AIC: 340.18
##
## Number of Fisher Scoring iterations: 4
```

```
#Obtaining the results expressed as a risk ratio (RR)

exp(coef(modelo_2))
```

```
## (Intercept)       AGE         SEX       Chr.dis      Help      Pol.Level
##   3.5268849   1.0023878   1.0000337   1.0161882   0.9883809   1.339314
##     Cog_Cap         NOM         DRP
##   1.0191102   0.9963955   1.0065799
```

```
confint(model_2)
```

```
## Waiting for profiling to be done...
```

```
##                    2.5 %      97.5 %
## (Intercept)  0.702191134 1.813761107
## AGE         -0.003759025 0.008615014
## Sex         -0.165904115 0.169631460
## Chr.dis     -0.027614459 0.059339077
## Help        -0.169219081 0.145355752
## Pol.level    0.195525235 0.389414119
## Cog_Cap     -0.063959936 0.100571587
## NOM         -0.045295360 0.037805830
## DRP         -0.023615210 0.036606992
```

```
# Assessing data normality

shapiro.test(residuals(modelo_2))
```

```
##
## Shapiro-Wilk normality test
##
## data:  residuals(modelo_2)
## W = 0.98309, p-value = 0.4168
```

```
# Graphical representation of the residuals for the model_2

oldpar<-par(mfrow =c(2,2))
plot(model_2)
```

Appendix 2

Abbreviation

Chr.dis: Chronic Diseases

Cop_Cap: Cognitive Capacity

NOM: Negative Outcomes Associated

DRP: Drugs-Related Problems

```
model_2 <- glm(model, family =quasipoisson (link=log), data = data)
anova(model_2)

## Analysis of Deviance Table
##
## Model: quasipoisson, link: log
##
## Response: Med
##
## Terms added sequentially (first to last)
##
##
##           Df Deviance Resid. Df Resid. Dev
## NULL                         74     86.476
## AGE        1    1.559        73     84.917
## Sex        1    3.471        72     81.446
## Chr.dis    1   33.992        71     47.454
## Help       1    0.005        70     47.449
## Pol.level  1   38.006        69      9.442
## Cog_Cap    1    0.229        68      9.214
## DRP        1    0.153        67      9.060
## NOM        1    0.029        66      9.031

summary(model_2)

##
## Call:
## glm(formula = model, family = quasipoisson(link = log), data = datos)
##
## Deviance Residuals:
##      Min        1Q    Median        3Q       Max
## -0.97385  -0.21893   0.00023   0.19935   0.88322
##
## Coefficients:
##              Estimate Std. Error t value Pr(>|t|)
## (Intercept)  1.260e+00  1.040e-01  12.124   <2e-16 ***
## AGE          2.385e-03  1.157e-03   2.061   0.0433 *
## Sex          3.374e-05  3.137e-02   0.001   0.9991
## Chr.dis      1.606e-02  8.134e-03   1.974   0.0525 .
## Help        -1.169e-02  2.942e-02  -0.397   0.6925
## Pol.level    2.922e-01  1.813e-02  16.113   <2e-16 ***
## Cog_Cap      1.893e-02  1.539e-02   1.230   0.2230
```

```
## DRP          6.558e-03  5.632e-03   1.164   0.2485
## NOM         -3.611e-03  7.772e-03  -0.465   0.6437
## ---
## Signif. codes:  0 '***' 0.001 '**' 0.01 '*' 0.05 '.' 0.1 ' ' 1
##
## (Dispersion parameter for quasipoisson family taken to be 0.1344583)
##
##     Null deviance: 86.4761  on 74  degrees of freedom
## Residual deviance:  9.0314  on 66  degrees of freedom
## AIC: NA
##
## Number of Fisher Scoring iterations: 4
```

```
# Obtaining the results expressed as a risk ratio (RR)

exp(coef(model_2))
```

```
## (Intercept)         AGE         SEX     Chr.dis       Help     Pol.level
##   3.5268849   1.0023878   1.0000337   1.0161882   0.9883809   1.3393143
##     Cap_Cog         DRP         NOM
##   1.0191102   1.0065799   0.9963955
```

```
confint(model_2)
```

```
## Waiting for profiling to be done...
```

```
##                     2.5 %      97.5 %
## (Intercept)  1.056322e+00 1.463853068
## AGE          1.224978e-04 0.004658968
## Sex         -6.120950e-02 0.061768359
## Crh.dis      9.029186e-05 0.031974029
## Help.       -6.938153e-02 0.045941561
## POL.level    2.566600e-01 0.327739384
## Cog_Cap     -1.131351e-02 0.049005590
## DRP         -4.489657e-03 0.017589527
## NOM         -1.886228e-02 0.011604325
```
